# Arthroscopic reverse remplissage combined with posterior Bankart repair: a report of 2 cases

**DOI:** 10.1016/j.xrrt.2025.02.009

**Published:** 2025-03-23

**Authors:** Yuki Yoshida, Takeshi Ikegami

**Affiliations:** Department of Orthopaedic Surgery, Fussa Hospital, Tokyo, Japan

**Keywords:** recurrent posterior dislocation, posterior shoulder instability, posterior Bankart repair, reverse Hill-Sachs lesion, subscapularis remplissage, reverse remplissage

## Introduction

Recurrent posterior shoulder dislocation with a reverse Hill-Sachs lesion is a rare and challenging condition. This report describes 2 cases successfully treated with arthroscopic reverse remplissage and posterior Bankart repair, demonstrating favorable outcomes without significant postoperative motion restrictions. A reverse Hill-Sachs lesion engages the glenoid rim during internal rotation, leading to recurrent dislocation. This can be effectively addressed by reverse remplissage, which anchors the subscapularis into the lesion. Additionally, posterior Bankart repair can be also performed efficiently viewed from the posterior portal. We outline the surgical technique and postoperative outcomes, emphasizing the efficacy of this combined approach.

## Case report

### Case 1

**Figure 1:**
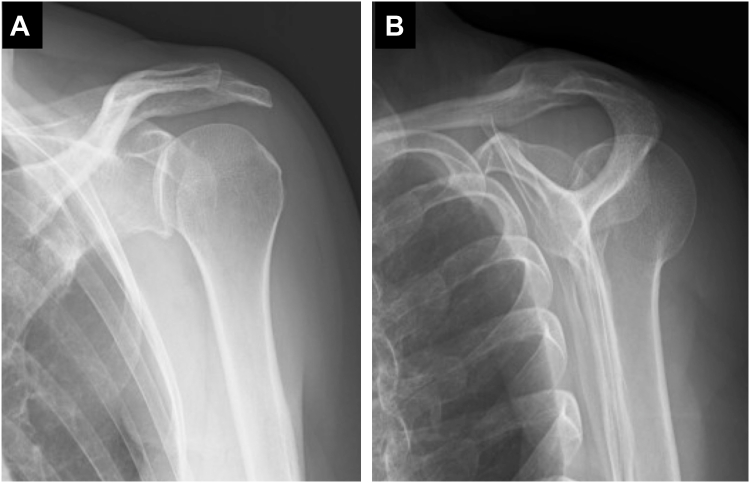
Initial radiographs of Case 1. (**A**) Anteroposterior view. (**B**) Scapula Y view.

**Figure 2:**
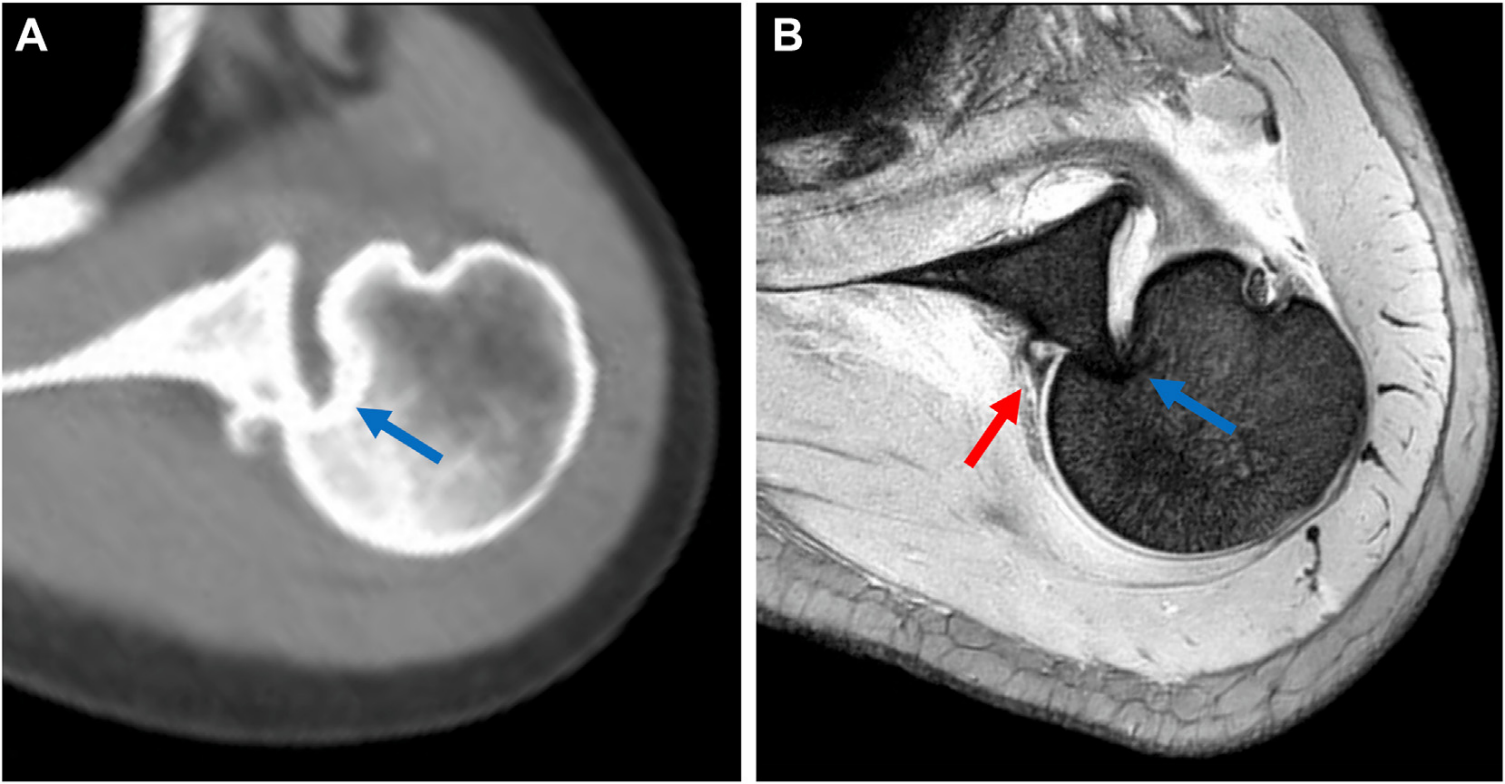
(**A**) CT and (**B**) T2 star–weighted MRI images of Case 1, obtained at a local hospital, revealed a Hill-Sachs lesion (*blue arrow*) and a posterior Bankart lesion (*red arrow*). *CT*, computed tomography; *MRI*, magnetic resonance imaging.

A 47-year-old female presented with left shoulder pain after falling from a bicycle and landing on the anterior aspect of her left shoulder. Diagnosed with a posterior dislocation, she underwent closed reduction attempts at a local hospital. Despite 3 reduction attempts, she experienced recurrent posterior dislocations while her shoulder was immobilized in a sling with internal rotation. Two weeks after the injury, due to the difficulty of conservative management, she was referred to our hospital for surgical intervention. Initial radiographs revealed a persistent posterior dislocation with the characteristic “lightbulb sign” on anteroposterior view (Fig. 1). Computed tomography showed a reverse Hill-Sachs lesion engaging the glenoid rim, involving approximately 25% of the humeral head articular surface.^3^ Magnetic resonance imaging confirmed a posterior Bankart lesion (Fig. 2). Given her persistent shoulder instability and imaging findings, surgical intervention was planned without attempting rereduction or further conservative treatment.

**Figure 3:**
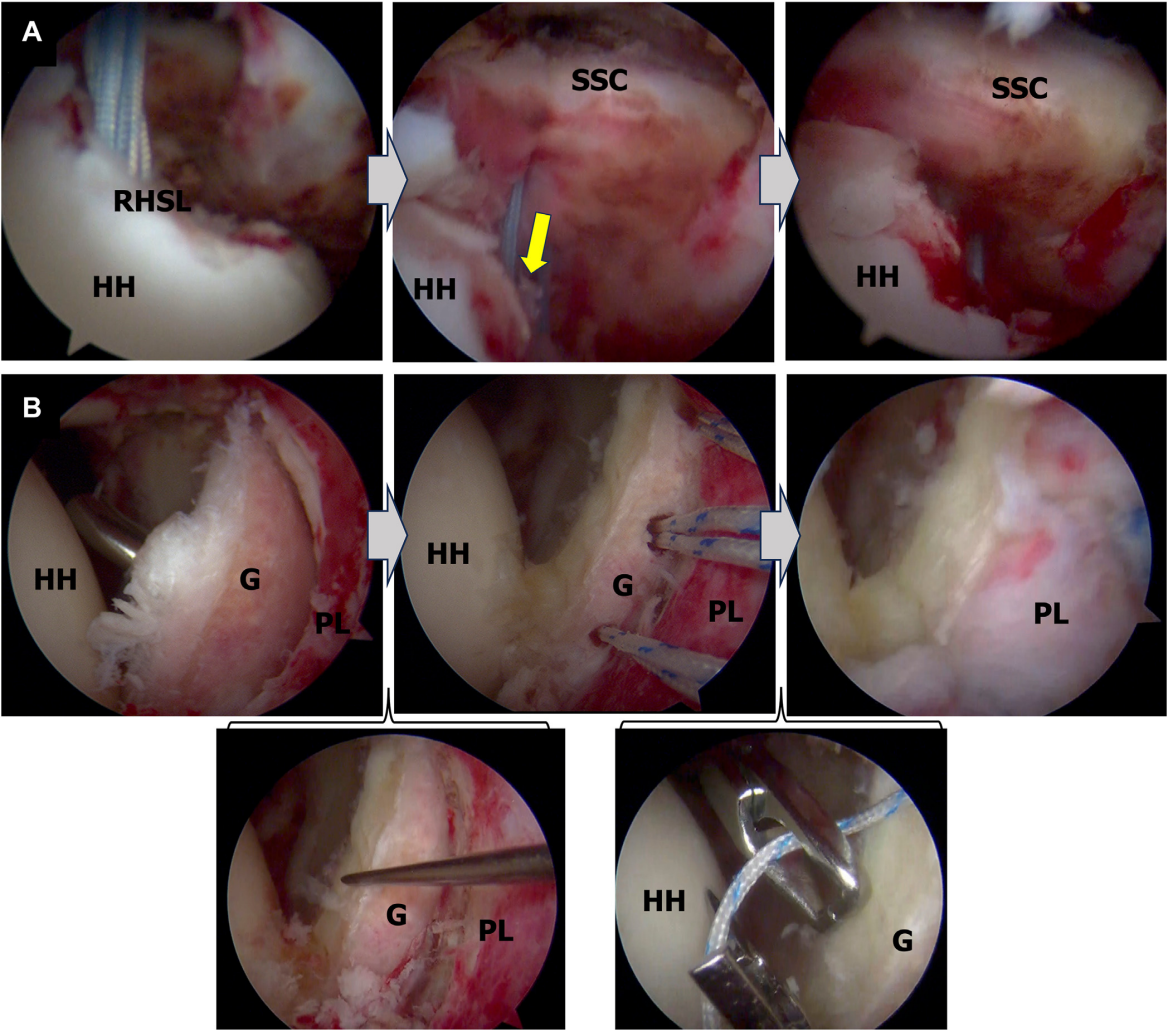
Intraoperative arthroscopic view of Case 1. (**A**) The reverse remplissage process as viewed from the posterior portal. Anchoring the subscapularis into the reverse Hill-Sachs lesion (*yellow arrow*). (**B**) The posterior Bankart repair sequence as viewed from the posterior portal. To ensure adequate access to the posterior glenoid, an 18-gauge spinal needle was used to create a posterior working portal (posterosuperior portal). The posterior labrum was threaded using a penetrator suture retriever and single sutured. *G*, glenoid; *HH*, humeral head; *PL*, posterior labral.

The surgery was planned to address both the reverse Hill-Sachs and posterior Bankart lesions by performing an arthroscopic reverse remplissage and posterior Bankart repair (Table I). The patient was positioned in the beach-chair position, and the surgical procedure began with diagnostic arthroscopy through a standard posterior portal. The reverse remplissage was performed first, as this maneuver moves the humeral head anteriorly. Performing reverse remplissage before posterior Bankart repair facilitates better access for subsequent posterior labral repair and allows for standardization shoulder instability management. Two 4.75-mm bioabsorbable anchors (HEALICOIL REGENESORB; Smith & Nephew ASD, Inc, Andover, MA, USA) were placed into the reverse Hill-Sachs lesion through the rotator interval (RI). The subscapularis was sutured using a penetrator, and transtendinous mattress sutures were passed through it following previously described techniques,^2^^,^^6^^,^^7^ without transposing its tendon attachment (Fig. 3, *A*). To minimize restriction, the subscapularis was sutured in external rotation. Following the reverse remplissage, posterior Bankart repair was performed with visualization from the posterior portal. To ensure adequate access to the posterior glenoid, an 18-gauge spinal needle was used to create a posterior working portal (posterosuperior portal). Three 1.8-mm all-suture anchors (Q-Fix; Smith and Nephew ASD, Inc, Andover, MA, USA) were placed at posterior glenoid rim. The posterior labrum was threaded using a penetrator suture retriever and single sutured (Fig. 3, *B*).

**Table I:** Surgical pearls and pitfalls.

	Pearl	Pitfall
Reverse remplissage	1. Place anchors through rotator interval	1. Placing anchors too medially can restrict range of motion
	2. Suture the subscapularis in external rotation to minimize motion restriction	2. Poor anchor placement may lead to inadequate filling of the defect
	3. Place sutures as laterally as possible on the subscapularis	3. Improper suture placement can cause tissue bunching
Posterior Bankart repair	1. Create posterosuperior portal with spinal needle guidance for optimal access	1. Inadequate labral mobilization can lead to poor tissue healing
	2. Maintain visualization from posterior portal throughout repair	2. Poor tissue tensioning can result in residual instability
	3. Ensure adequate tissue mobilization before repair	3. Limited access due to improper portal placement

**Figure 4:**
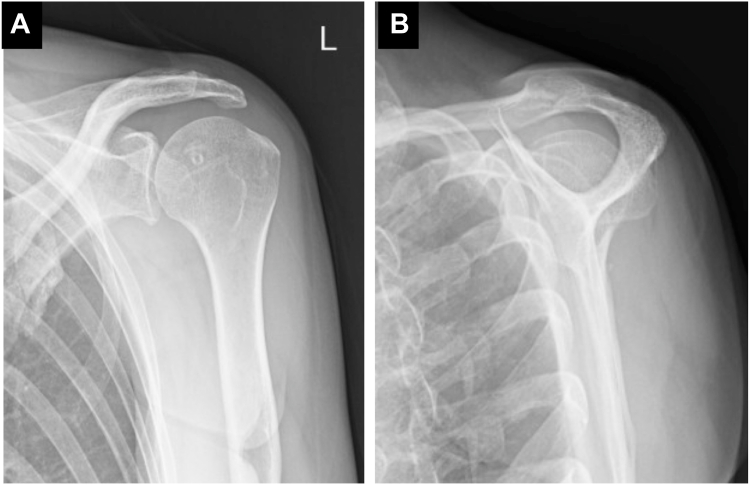
Postoperative radiographs of Case 1. (**A**) Anteroposterior view. (**B**) Scapula Y view.

**Figure 5:**
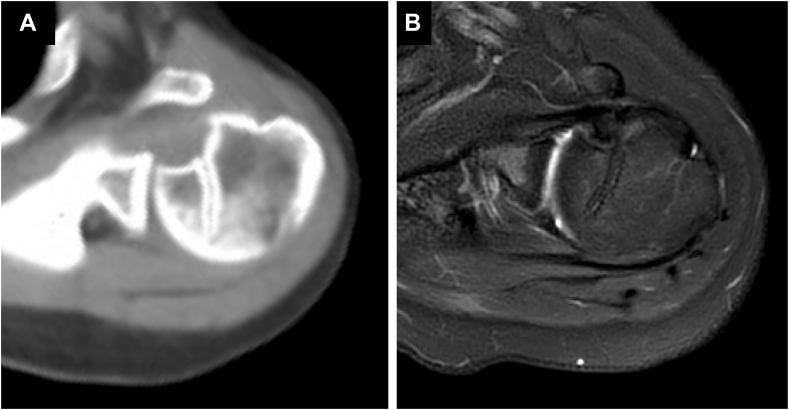
(**A**) Postoperative CT and (**B**) T2-weighted fat-suppressed MRI images of Case 1. CT scans were obtained 3 months after surgery, and MRI scans were acquired 6 months after surgery, confirming maintained reduction and successful attachment of the subscapularis to the reverse Hill-Sachs lesion. *CT*, computed tomography; *MRI*, magnetic resonance imaging.

**Figure 6:**
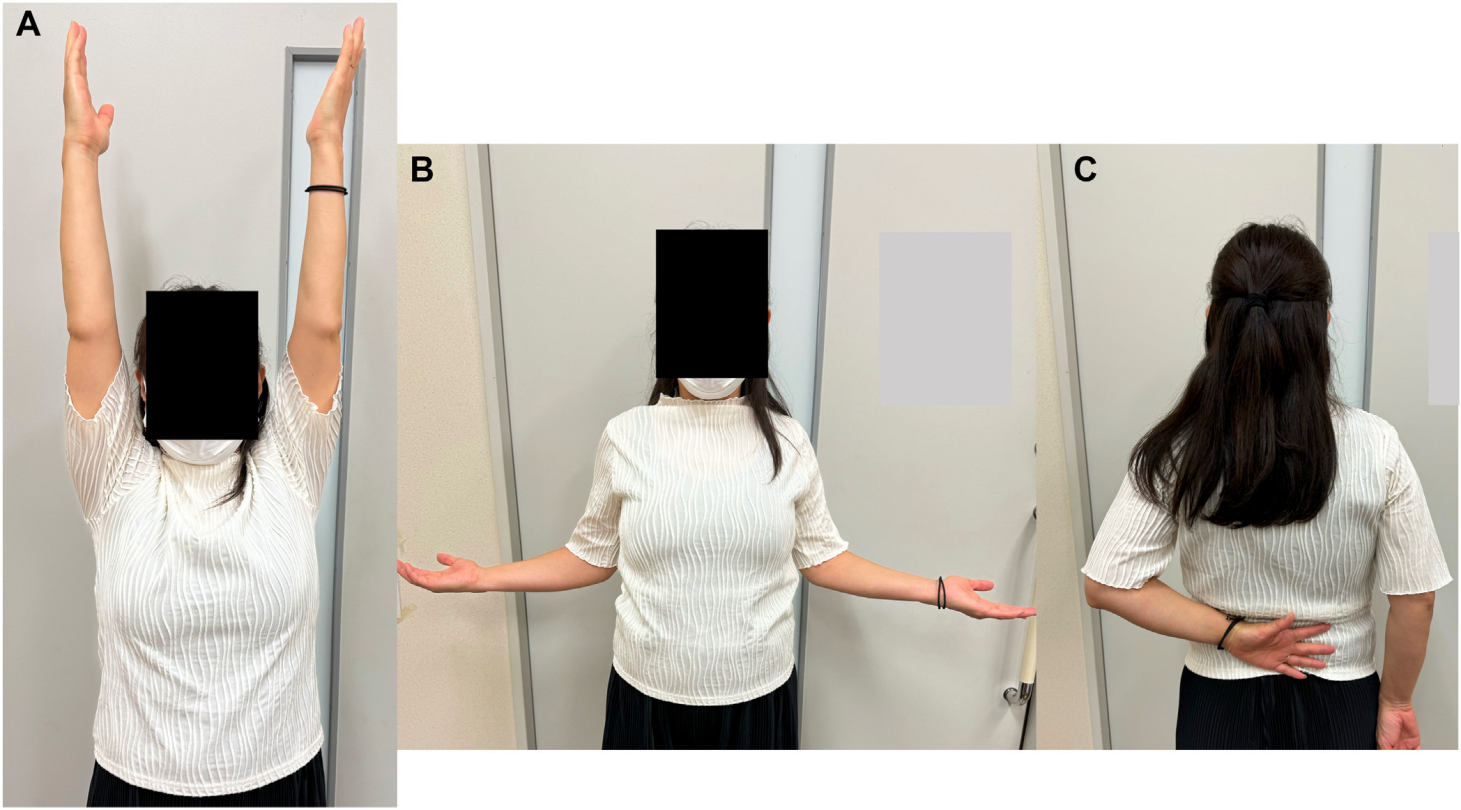
Range of motion of Case 1 at 6 months post operation. (**A**) Elevation. (**B**) External rotation. (**C**) Internal rotation.

Postoperatively, the patient wore a sling in a neutral rotation for 4 weeks. Passive elevation was permitted starting 2 weeks after surgery, with internal rotation avoided. The sling was removed at 4 weeks postsurgery, allowing for active elevation and external rotation, while internal rotation was permitted starting at 6 weeks postsurgery. Follow-up imaging confirmed maintained reduction (Fig. 4) and successful subscapularis tendon attachment to the reverse Hill-Sachs lesion (Fig. 5). At 6-month follow-up, the patient demonstrated excellent outcomes with an American Shoulder and Elbow Surgeons score of 97, satisfactory range of motion (170° elevation, 70° external rotation, internal rotation to Th10 vertebral level), and complete return to daily activities without shoulder pain or instability (Fig. 6).

### Case 2

**Figure 7:**
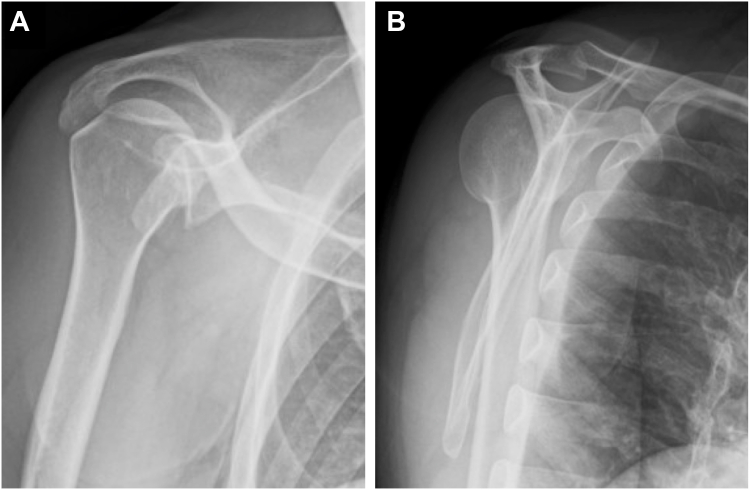
Initial radiographs of Case 2. (**A**) Anteroposterior view. (**B**) Scapula Y view.

**Figure 8:**
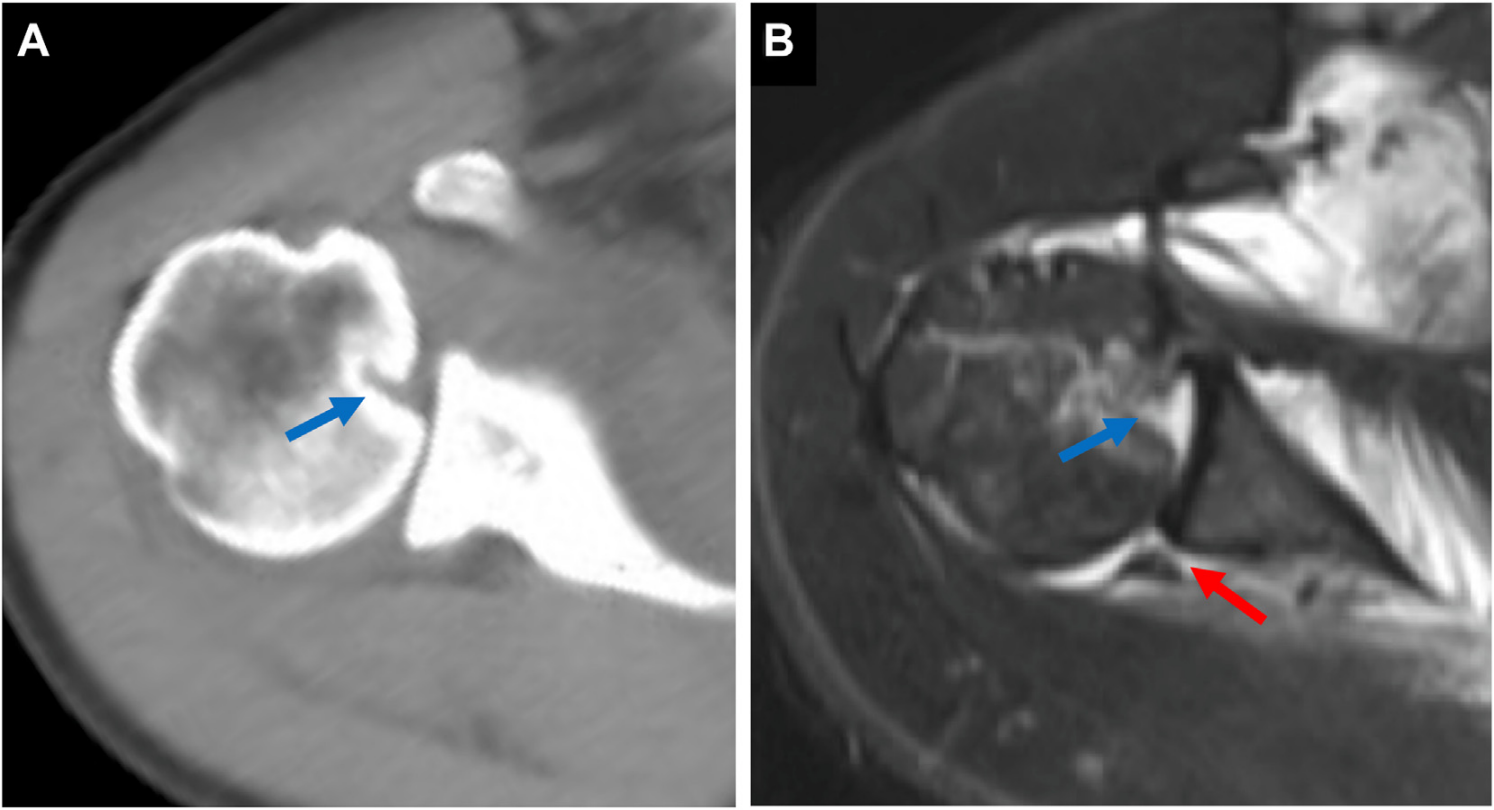
(**A**) CT and (**B**) T2-weighted fat-suppressed MRI images of Case 2, obtained at a local hospital, revealed a reverse Hill-Sachs lesion (*blue arrow*) and a posterior Bankart lesion (*red arrow*). *CT*, computed tomography; *MRI*, magnetic resonance imaging.

A 56-year-old male sustained a right shoulder injury after falling while intoxicated and was initially diagnosed with a posterior shoulder dislocation. He underwent a closed reduction at a local hospital but experienced recurrent posterior dislocations 2 weeks after the injury while immobilized in a sling with internal rotation. Consequently, he was referred to our hospital for surgical intervention. Although the reverse Hill-Sachs lesion was smaller than that in Case 1, similar imaging and physical findings were observed (Figure 7, Figure 8 and Figure 7, Figure 8). Given his persistent shoulder instability and imaging findings, surgical intervention was planned without further attempts at conservative treatment, such as immobilization in a sling with neutral or external rotation.

**Figure 9:**
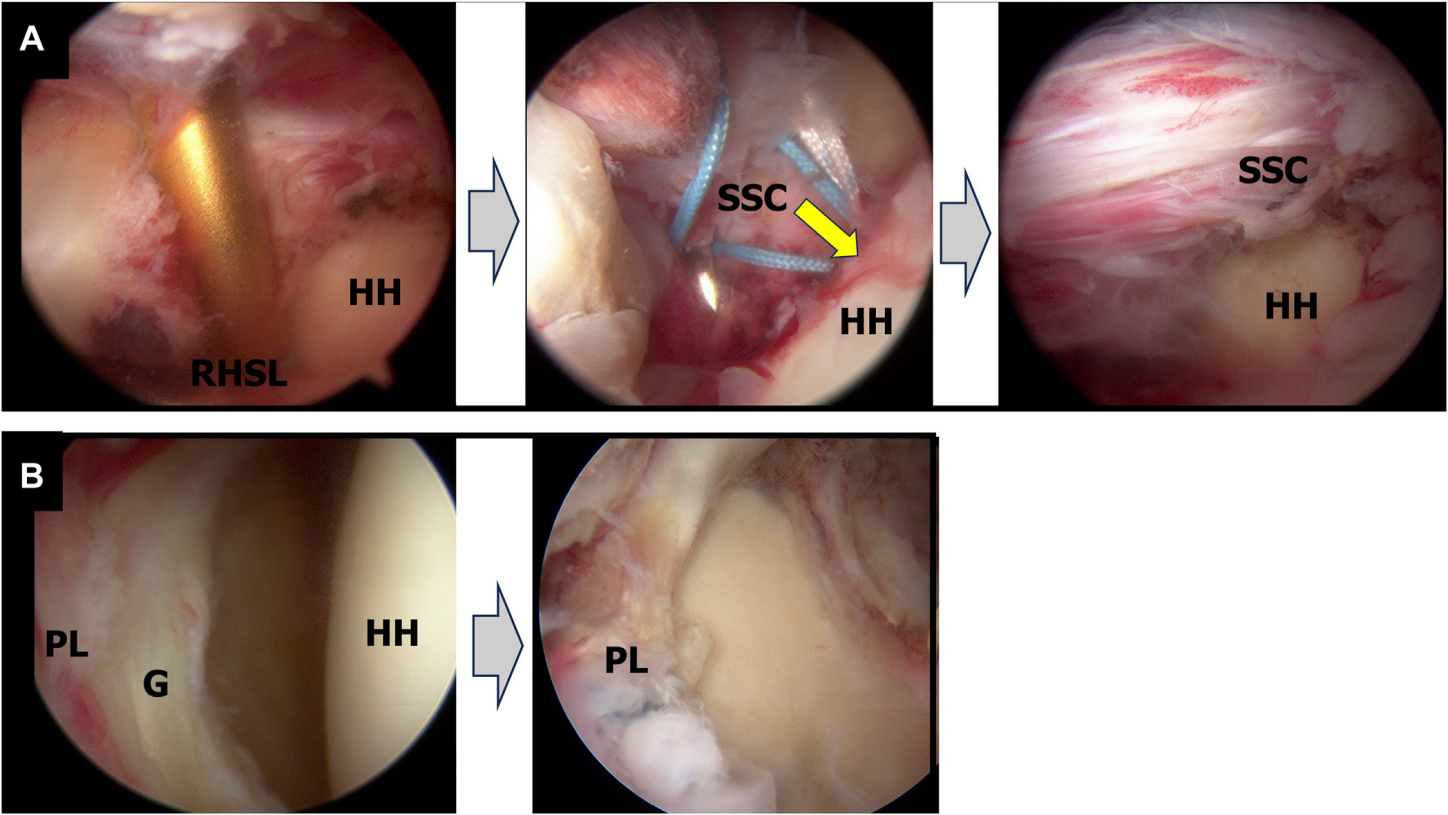
Intraoperative arthroscopic view of Case 2. (**A**) The reverse remplissage process as viewed from the posterior portal. Anchoring the subscapularis into the reverse Hill-Sachs lesion (*yellow arrow*). (**B**) Before and after posterior Bankart repair viewed from the posterior portal. *G*, glenoid; *HH*, humeral head; *PL*, posterior labral.

**Figure 10:**
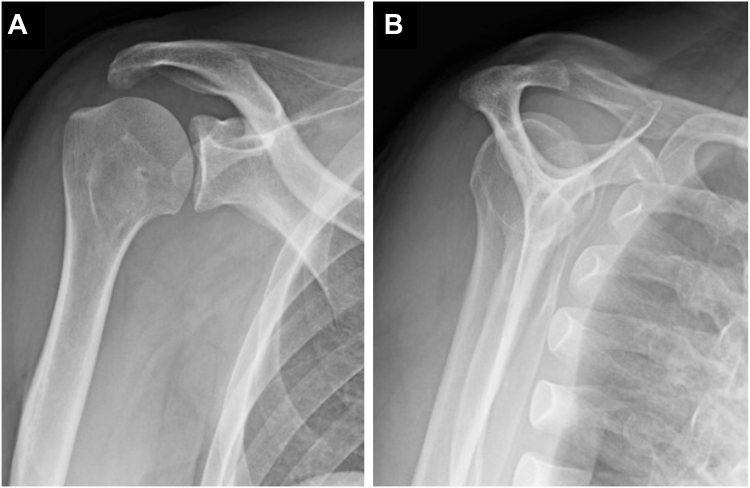
Postoperative radiographs of Case 2. (**A**) Anteroposterior view. (**B**) Scapula Y view.

**Figure 11:**
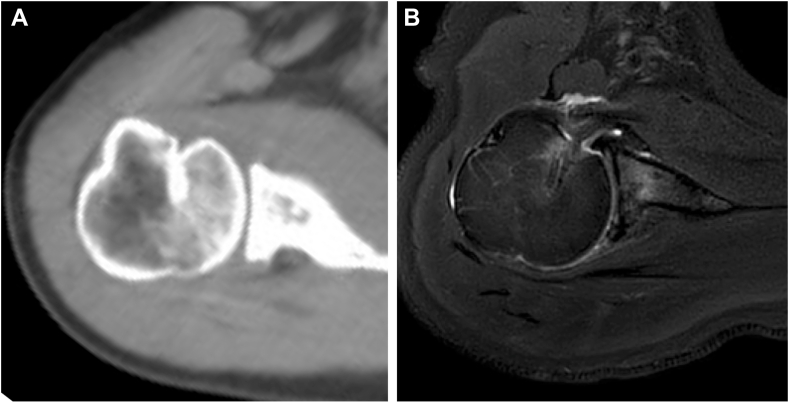
(**A**) Postoperative CT and (**B**) T2-weighted fat-suppressed MRI images of Case 2. CT scans were obtained 3 months after surgery, and MRI scans were acquired 6 months after surgery, confirming maintained reduction and successful attachment of the subscapularis to the reverse Hill-Sachs lesion. *CT*, computed tomography; *MRI*, magnetic resonance imaging.

**Figure 12:**
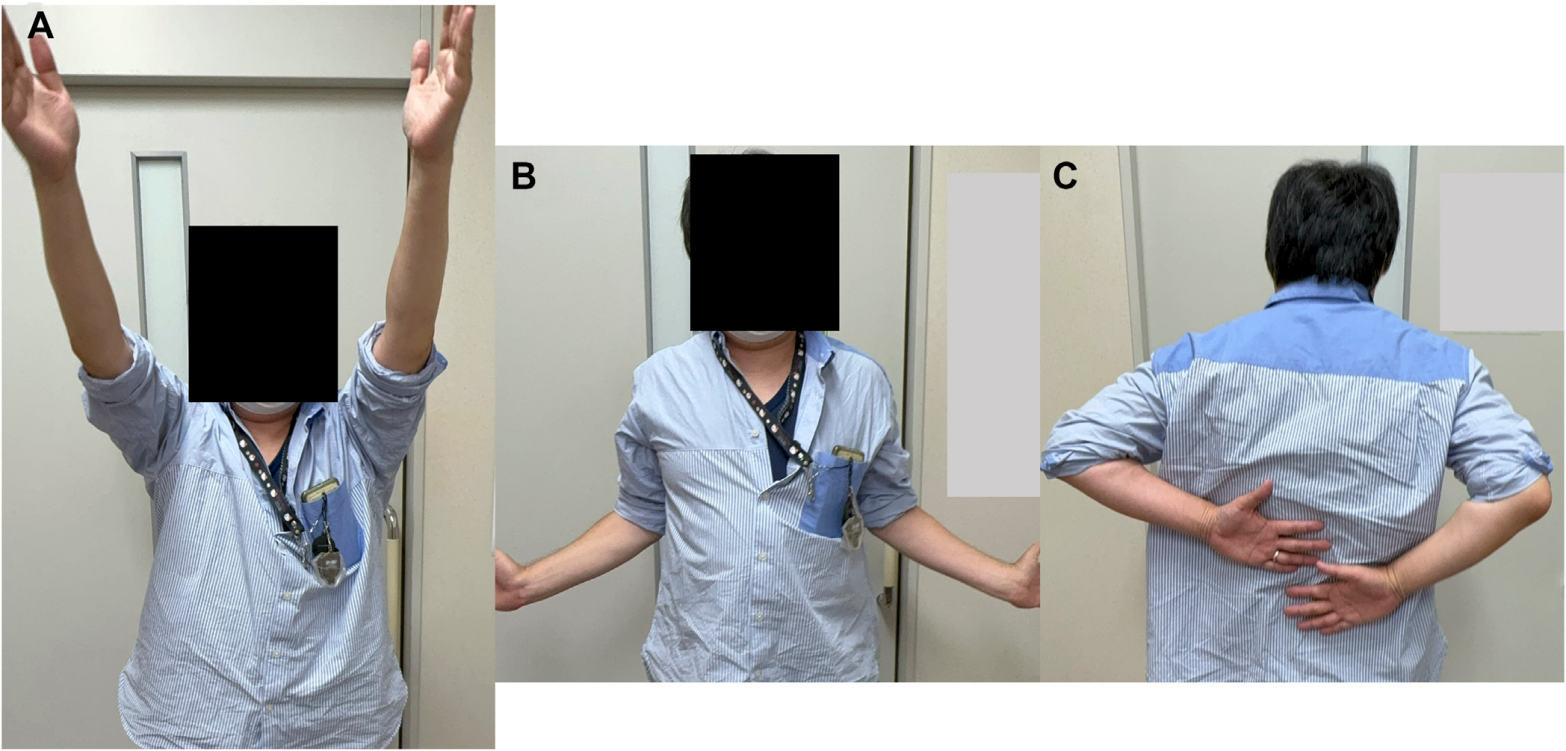
Range of motion of Case 2 at 6 months post operation. (**A**) Elevation. (**B**) External rotation. (**C**) Internal rotation.

The surgical approach mirrored that of Case 1, combining reverse remplissage and posterior Bankart repair (Fig. 9). Rehabilitation followed the same protocol as in Case 1, leading to a stable reduction and favorable outcomes (Figure 10, Figure 11 and Figure 10, Figure 11). Six-month follow-up showed similar excellent results with an American Shoulder and Elbow Surgeons score of 95, satisfactory range of motion (170° elevation, 60° external rotation, internal rotation to Th10 vertebral level), and complete return to daily activities without shoulder pain or instability (Fig. 12).

## Discussion

Posterior shoulder dislocations are rare injuries, often resulting from trauma such as a direct impact to the humeral head, a fall onto an outstretched arm, or a motor vehicle accident.^3^^,^^10^^,^^12^ They can also frequently occur secondary to epileptic seizures or electrical injuries. Traumatic posterior shoulder dislocations can result in impression on the anterior surface of the humeral head, leading to a reverse Hill-Sachs lesion. This bony defect can engage with the posterior glenoid rim, creating abnormal joint mechanics that cause mechanical symptoms, pain, or recurrent shoulder dislocations.^1^^,^^11^ In our cases of posterior shoulder dislocation with a reverse Hill-Sachs lesion, immobilization in internal rotation increases the risk of redislocation due to lesion engagement. These cases highlight the importance of avoiding internal rotation postreduction to prevent recurrence.

Conservative treatment yields positive outcomes in most patients; however, 65%-80% of cases experience recurrent posterior dislocations.^1^ Surgical intervention is indicated for recurrent instability caused by an engaging reverse Hill-Sachs lesion, not only to stabilize the shoulder but also to prevent progressive joint destruction and early onset osteoarthritis. Given the recurrent nature of posterior shoulder dislocations in our cases, we chose surgical management.

Several surgical techniques address posterior shoulder instability beyond posterior Bankart repair, including capsular shift and RI closure, which stabilize the joint by modifying the anterior capsule.^14^, ^15^, ^16^ To manage the reverse Hill-Sachs lesion, McLaughlin procedure^10^ involving the transfer of the subscapularis tendon into the anterior humeral defect has been the traditional treatment for these lesions. Krackhardt et al.^6^ later developed the first all-arthroscopic modification, commonly known as reverse remplissage^2^^,^^7^^,^^9^ or subscapularis remplissage,^8^^,^^13^ which fixes the subscapularis tendon to the reverse Hill-Sachs lesion without transposing its attachment. As opposed to the traditional McLaughlin procedure, there is no transposition of the subscapularis tendon attachment. We adopted arthroscopic reverse remplissage,^2^^,^^7^^,^^9^ suturing the subscapularis without detachment or transposition of the subscapularis tendon attachment.

To compared with the standard remplissage for anterior dislocation with a Hill-Sachs lesion,^5^ the infraspinatus is sutured to the lesion under arthroscopic viewing of both the bursal and articular sides. In reverse remplissage, however, an anchor is inserted through the RI, allowing direct subscapularis suturing without changing the view of the articular side, thus simplifying the procedure compared to standard remplissage. Although there are concerns about limiting the range of motion in internal and external rotation, some studies have reported detaching the superior edge of the subscapularis to allow secure suturing into the defect while minimizing motion restriction.^17^ In our case, we placed sutures as laterally as possible on the subscapularis in external rotation, avoiding significant motion limitations.

In Case 2, the reverse Hill-Sachs lesion was relatively small; therefore, arthroscopic posterior Bankart repair alone might have been sufficient. Good clinical outcomes have been reported with the combination of arthroscopic remplissage and Bankart repair for anterior instability,^4^ and this approach is believed to provide the same benefits for posterior instability. In our procedure, both reverse remplissage and posterior Bankart repair were performed easily while maintaining an articular side view through the posterior portal. The efficacy of arthroscopic reverse remplissage combined with posterior Bankart repair was demonstrated in Case 1. The same combined arthroscopic approach was chosen for Case 2, which also resulted in a favorable outcome without limitations in internal or external rotation.

This combined arthroscopic approach provides a minimally invasive, technically straightforward, and effective method for managing reverse Hill-Sachs lesions in recurrent posterior shoulder dislocations. While there is a potential risk of limited range of motion, no significant postoperative restrictions, including internal rotation, were noted. This approach has shown favorable outcomes without complications.

## Conclusion

We present 2 cases of recurrent posterior shoulder dislocation with reverse Hill-Sachs lesions. To prevent recurrence, immobilization in internal rotation should be avoided after reduction. Arthroscopic posterior Bankart repair combined with reverse remplissage offers a minimally invasive, technically straightforward approach for managing reverse Hill-Sachs lesions, resulting in favorable clinical outcomes without significant postoperative motion restrictions.

## Acknowledgments

The authors express their gratitude to Atsushi Yoshida, Yoshiko Yoshida, Shoko Yoshida, Yuma Yoshida, Runa Yoshida, and the orthopedic surgeons at Fussa Hospital for their invaluable support and cooperation.

## Disclaimers:

Funding: No funding was disclosed by the authors.

Conflicts of interest: The authors, their immediate families, and any research foundation with which they are affiliated have not received any financial payments or other benefits from any commercial entity related to the subject of this article.

Patient consent: Obtained.
